# The pharmacokinetics and pharmacodynamics of alogliptin in children, adolescents, and adults with type 2 diabetes mellitus

**DOI:** 10.1007/s00228-016-2175-1

**Published:** 2016-12-20

**Authors:** Caroline Dudkowski, Max Tsai, Jie Liu, Zhen Zhao, Eric Schmidt, Jeannie Xie

**Affiliations:** 1 Takeda Development Center Americas, Inc., One Takeda Parkway, Deerfield, IL 60015 USA; 2 Takeda Pharmaceuticals U.S.A., Inc., One Takeda Parkway, Deerfield, IL 60015 USA

**Keywords:** Alogliptin, Pharmacokinetics, Pharmacodynamics, Pediatric patients, DPP-4 inhibition

## Abstract

**Purpose:**

The aim of this study is to determine the pharmacokinetics (PK) and pharmacodynamics (PD) of a single 12.5- or 25-mg dose of alogliptin, a dipeptidyl peptidase-4 (DPP-4) inhibitor, in pediatric (children and adolescents) and adult subjects with type 2 diabetes mellitus (T2DM).

**Methods:**

A randomized, open-label, multicenter study was conducted in pediatric and adult subjects. Subjects in two pediatric groups (children and adolescents) were randomized 1:1 to receive a single oral dose of alogliptin 12.5 or 25 mg, respectively; all gender- and race-matched adult subjects received alogliptin 25 mg. Blood and urine samples were collected at prespecified time points for PK/PD analyses. A PK/PD model was developed using data from the study for steady-state simulations. Safety was also assessed.

**Results:**

In pediatric subjects receiving the 25-mg dose, the mean alogliptin peak plasma concentrations (C_max_) and AUC_0-inf_ values were 26 and 23% lower, respectively, than in adults receiving the 25-mg dose, but maximum observed DPP-4 inhibition effect (E_max_) and AUEC_0–24_ values were similar to those in adults. In pediatric subjects receiving the 12.5-mg dose, the mean alogliptin C_max_ and AUC_0-inf_ values were 58 and 54% lower, respectively, than those in adults, hence E_max_ and AUEC_0–24_ values were also lower by 11 and 17%, respectively. The PK/PD model simulated data were consistent with study results. No safety concern was found.

**Conclusions:**

A 25-mg dose of alogliptin in pediatric subjects achieved alogliptin exposures and DPP-4 inhibition similar to those in adult T2DM patients without safety concerns; therefore, this dose is recommended for a pediatric phase 3 trial.

**Electronic supplementary material:**

The online version of this article (doi:10.1007/s00228-016-2175-1) contains supplementary material, which is available to authorized users.

## Introduction

Type 2 diabetes mellitus (T2DM), though historically recognized as a disease of adulthood, has become increasingly common among children [1]. From 2001 to 2009, the prevalence of T2DM among children aged between 10 and 19 in the USA rose from 0.34 per 1000 to 0.46 per 1000, an overall increase of 30.5% [2]. The prevalence is significantly higher in Native Americans and African Americans than in other races [2].

Dipeptidyl peptidase-4 (DPP-4) is the primary enzyme involved in the rapid in vivo degradation of the incretin hormones, which play an important role in the pathogenesis of T2DM [3]. The incretin hormones include glucagon-like peptide-1 (GLP-1) and glucose-dependent insulinotropic peptide (GIP), two peptides that exert major glucoregulatory functions [3]. Released upon nutrient ingestion, these peptides stimulate glucose-dependent insulin secretion and promote beta-cell proliferation and cytoprotection [3, 4]. In patients with T2DM, GIP is produced at normal levels with impaired glucose-lowering action, while GLP-1 retains its glucoregulatory activity despite diminished secretion [5, 6]. Thus, inhibition of DPP-4 activity increases plasma GLP-1 levels, which in turn augments insulin secretion and reduces blood glucose levels [7, 8].

Alogliptin is a DPP-4 inhibitor indicated as an adjunct to diet and exercise to improve glycemic control in adults with T2DM [9]. Clinical studies have demonstrated that alogliptin produces rapid and sustained DPP-4 inhibition, improves both postprandial and fasting plasma glucose, and reduces glycated hemoglobin levels by approximately 0.5 to 1% after 12 to 26 weeks of treatment in adult T2DM patients [10–13]. Combined therapy using alogliptin with other non-DPP-4 inhibitor antidiabetic agents provides significantly better glycemic control than monotherapy with any of these agents alone [13–19].

The objectives of this study were to determine the pharmacokinetic (PK) and pharmacodynamic (PD) profiles of a single dose of alogliptin 12.5 and 25 mg in pediatric (ages 10–17) subjects with T2DM, and compare the pediatric PK/PD profiles with those in adult subjects with T2DM.

## Methods

### Study design

This phase 1, randomized, open-label, single-dose, multicenter study (NCT00957268) was conducted in compliance with the institutional review board regulations stated in the US Code of Federal Regulations, Good Clinical Practice (GCP) regulations and guidelines; the ethical principles originated from the Declaration of Helsinki, the International Conference on Harmonization E6 GCP guidance, and all applicable local regulations. Signed informed consent forms and assent (if applicable) or certified translations of these forms (if applicable) were obtained from each subject or the subject’s legally acceptable representative before undergoing any study procedures.

The study enrolled male and female T2DM patients who were age-stratified into group 1 (children age 10 to <14 years), group 2 (adolescents age 14 to <18 years), and group 3 (gender- and race-matched adults age 18–65 years). A detailed description of subject enrollment criteria is available in supplemental materials.

A total of 24 pediatric subjects and 22 gender- and race-matched adult subjects were enrolled in the study. Since T2DM is uncommon among children, there were significant enrollment challenges, particularly for subjects below 14 years of age. As such, the number of enrolled subjects was based on review of past PK studies of a similar nature and extensive consultation of regulatory agencies. The final sample size was a minimum number of subjects deemed sufficient to meet study objectives. Statistical analysis was not performed to determine the sample size.

The duration of the study was approximately 47 days, consisting of screening (days −28 to −2), check-in (day −1), a treatment period (days 1–4), and a follow-up phone call at 14 ± 1 days after study exit. Following screening for eligibility, enrolled subjects were admitted to the clinic on day −1. On the first day of the treatment period, subjects in each pediatric group (group 1 and group 2) were randomized in a 1:1 ratio to receive a single, oral dose of alogliptin 12.5 or 25 mg, respectively; all adult subjects (group 3) received a single dose of alogliptin 25 mg. The study doses (12.5 and 25 mg) were selected based on the results of phase 3 studies in adults. Both dose levels were well tolerated in healthy adult subjects and adult subjects with T2DM. A single dose was used because alogliptin exhibits linear kinetic properties. The study drug was administered after an overnight fast of at least 8 h. Subjects were released from the clinic in the evening on day 2 after the completion of all procedures and returned to the clinic in the morning on days 3 and 4 for outpatient visits. Approximately 14 days (14 ± 1 days) after the final visit (study exit), subjects received a follow-up phone call to check for adverse events (AEs)/serious AEs and concomitant medication uses that had occurred since study exit.

### PK/PD bioanalytical analysis

For alogliptin concentration measurements, blood samples were collected 1 h before dosing and 1, 2, 4, 8, 12, 16, 24, 48, and 72 h after dosing; urine samples were collected from −12 to 0 h predose, and from 0 to 4, 4 to 8, 8 to 12, 12 to 24, and 24 to 36 h postdose. Alogliptin plasma and urine concentrations were measured using liquid chromatography with tandem mass spectrometry detection. Quantification of sample alogliptin levels was performed according to calibration standards under strict quality controls (QC). A detailed description of assay precision and accuracy is available in supplemental materials.

For DPP-4 inhibition measurements, blood samples were collected within 1 h before dosing and 2, 4, 8, 12, and 24 h postdose. Sample DPP-4 inhibition was determined by a fluorogenic method in 96-well plates under strict QCs. A detailed description of assay precision and accuracy is available in supplemental materials.

### Assessments

PK parameters were derived using noncompartmental methods with WinNonlin Enterprise, Version 6.3 (Pharsight Corp., Cary, NC). The following plasma PK parameters were evaluated: maximum observed plasma concentration (C_max_), time to reach C_max_ (T_max_), terminal elimination half-life (T_1/2_), area under the plasma concentration-time curve from time 0 to infinity (AUC_0-inf_), apparent clearance after oral administration (CL/F), and apparent volume of distribution (Vz/F). Evaluated urine PK parameters included renal clearance from 0 to 24 h postdose (CLr) and fraction of drug excreted in urine from 0 to 36 h postdose (Fe).

PD parameters and individual DPP-4 inhibition were generated using WinNonlin Enterprise, Version 6.3. The following PD parameters were evaluated: area under the plasma effect-time curve from time 0 to 24 h postdose (AUEC_0–24_), maximum observed effect (E_max_), time to reach E_max_ (time to E_max_), and observed effect at 24 h postdose (E_24_).

The primary endpoints included the following plasma PK parameters for alogliptin: C_max_, T_max_, T_1/2_, and AUC_0-inf_; the secondary endpoints included the following PD parameters for DPP-4 inhibition: AUEC_0–24_, E_max_, time to E_max_, and E_24_. Additional endpoints included the following plasma and urine PK parameters for alogliptin: CL/F, Vz/F, CLr, and Fe. Safety measurements were incidence of AEs, clinical laboratory test results (hematology, serum chemistry, urinalysis, fasting glucose, and blood glucose monitoring), vital sign measurements, 12-lead electrocardiogram (ECG) results, and physical examination findings.

### Statistical analysis

Descriptive statistics (*N*, mean, standard deviation [SD], % coefficient of variation, median, minimum, and maximum) were used to summarize plasma and urine PK and plasma PD parameters. Descriptive statistics were also used to summarize AEs and markedly abnormal vital sign and ECG results (*N* and % of subjects), as well as clinical laboratory test results (hematology and serum chemistry), vital sign results, body weights, and ECG results (*N*, mean, SD, median, minimum, and maximum).

### PK/PD modeling and simulations

Using adult and pediatric data from this study, an alogliptin PK/PD model was developed to characterize the time course of alogliptin concentrations and DPP-4 inhibition. To bridge the adult and pediatric PK, the PK model included weight as an initial covariate on clearance and volume of distribution parameters, as shown in the following equation:

$$ {P}_i={P}_{\mathrm{pop}}\cdot {\left(\frac{WT_i}{WT_{\mathrm{reference}}}\right)}^b\cdot {e}^{\upeta_{\mathrm{i}}} $$

where *P*_*i*_ is the individual PK parameter, *P*_pop_ is the population PK parameter, *WT*_*i*_ is the individual body weight, *WT*_reference_ is the reference body weight of 70 kg, *b* represents a power function describing the relationship between weight and the PK parameter (*b* = 0.75 for CL/F and *b* = 1 for Vz/F), and *ƞ*_*i*_ represents the intersubject variability with a mean = 0 and variance = ω^2^. Other covariates, age (year), estimated glomerular filtration rate (mL/min/1.73m^2^), race, and sex, were also investigated as potential predicators of PK and PD parameters, CL/F, Vz/F, absorption rate constant, and half-maximal concentration. Covariate analyses were performed using univariate evaluation (*p* < 0.05, ΔOFV ≥3.84) followed by a stepwise backward elimination approach (*p* < 0.001, ΔOFV ≥10.83). The relationship between alogliptin concentrations and DPP-4 inhibition was described using a sigmoid E_max_ function in a simultaneous PK/PD model as follows:

$$ E={E}_0+\frac{E_{\max }*{\mathrm{Conc}}^{\gamma }}{{EC_{50}}^{\gamma }+{\mathrm{Conc}}^{\gamma }}+\varepsilon $$

where *E* is DPP-4 inhibition, *E*_0_ is baseline response (fixed to 0), E_max_ is the predicted maximal response, Conc is predicted alogliptin concentration, EC_50_ is the concentration resulting in half-maximal response, *γ* is shape factor, and *ε* represents the residual error with a mean = 0 and variance = σ^2^; separate residual error terms were used for PK (alogliptin concentration) and PD (DPP-4 inhibition) measurements.

Model-based simulations were performed to project the time course of alogliptin concentrations and DPP-4 inhibition following repeated dosing of alogliptin 12.5 and 25 mg in pediatric and adult populations. The simulations were based on 100 hypothetical subjects in each pediatric or adult group. For both groups, each subject’s body weight was sampled from a log-normal distribution of body weight centered around 95 kg (approximate study average), as body weights overlapped considerably between pediatric and adult subjects. The simulated data were summarized using noncompartmental methods (e.g., *C*_max_, area under the plasma concentration-time curve from time 0 to the end of the dosing period [AUC_0-tau_], E_24_).

## Results

### Subject baseline characteristics

A total of 126 subjects were screened, among whom 46 subjects were enrolled in the study and received the study drug. Reasons for screening failure included not meeting entrance criteria (66 subjects), voluntary withdrawal (4 subjects), lost to follow-up (1 subject), and other (9 subjects). Of the enrolled subjects, 9 were in group 1 (age 10 to <14 years) with 5 receiving alogliptin 12.5 mg and 4 receiving 25 mg; 15 were in group 2 (age 14 to < 18 years) with 8 receiving alogliptin 12.5 mg and 7 receiving 25 mg; and 22 were in group 3 (age ≥18 years) receiving alogliptin 25 mg. One of the 46 enrolled subjects (group 2, alogliptin 12.5 mg) discontinued on day 1 after receiving the study drug because blood samples could not be drawn through the intravenous line, leaving 45 subjects who completed all study visits.

Demographic information and baseline characteristics of study participants are shown in Table 1. Most subjects were female and black or African American. Within each pediatric group (group 1 and group 2), the mean age was similar between the two dose levels. The shortest duration of T2DM was 53 days. Fifteen of 24 (62.5%) pediatric subjects and 17 of 22 (77.3%) adult subjects had medical history conditions, primarily in the system organ class of surgical and medical procedures. Almost all subjects had concurrent medical conditions, primarily conditions associated with T2DM, such as obesity, hyperlipidemia, and hypertension. Twenty of the 24 pediatric subjects and 19 of the 22 adult subjects used concomitant metformin, and 18 of the 24 pediatric subjects and 19 of the 22 adult subjects used other concomitant medications, primarily medications used to treat hypertension or asthma/allergies.

**Table 1:** Demographic information and baseline characteristics of study participants

	Group 1, 10 to <14 years		Group 2, 14 to < 18 years		Group 3, adults
Characteristics	Alogliptin 12.5 mg *N* = 5	Alogliptin 25 mg *N* = 4	Alogliptin 12.5 mg *N* = 8	Alogliptin 25 mg *N* = 7	Alogliptin 25 mg *N* = 22
Age (years) Mean (SD)	12.4 (0.89)	12.0 (0.82)	15.4 (0.92)	15.1 (0.69)	51.3 (8.24)
Gender, *n* (%) Male Female	1 (20.0) 4 (80.0)	1 (25.0) 3 (75.0)	2 (25.0) 6 (75.0)	2 (28.6) 5 (71.4)	6 (27.3) 16 (72.7)
Race, *n* (%) Black or African American White	5 (100.0) 0 (0.0)	3 (75.0) 1 (25.0)	4 (50.0) 4 (50.0)	5 (71.4) 2 (28.6)	15 (68.2) 7 (31.8)
Ethnicity, *n* (%) Hispanic or Latino Not Hispanic or Latino	0 (0.0) 5 (100.0)	1 (25.0) 3 (75.0)	1 (12.5) 7 (87.5)	1 (14.3) 6 (85.7)	4 (18.2) 18 (81.8)
Weight (kg) Mean (SD)	86.62 (13.98)	98.90 (11.96)	116.28 (33.16)	103.71 (17.10)	92.25 (16.45)
BMI (kg/m^2^) Mean (SD)	33.22 (4.62)	36.16 (3.42)	40.92 (9.19)	36.46 (6.76)	32.84 (4.49)
CrCl^a^ Mean (SD)	111.46 (11.53)	114.84 (30.09)	124.88 (23.78)	112.63 (30.75)	87.69 (21.50)
Metformin (mg) Mean (SD)	1000 (612.4)^b^	1667 (577.4)^c^	1583 (664.6)^d^	1300 (670.8)^e^	1346 (591.1)^f^

*BMI*, body mass index; *CrCl*, creatinine clearance; *SD*, standard deviation

^a^ *CrCl* is calculated as mL/min/1.73 m^2^ in pediatric subjects and mL/min in adult subjects;

^b^ *N* = 5

^c^ *N* = 3

^d^ *N* = 6

^e^ *N* = 5

^f^ *N* = 13

### PK data

**Fig. 1:**
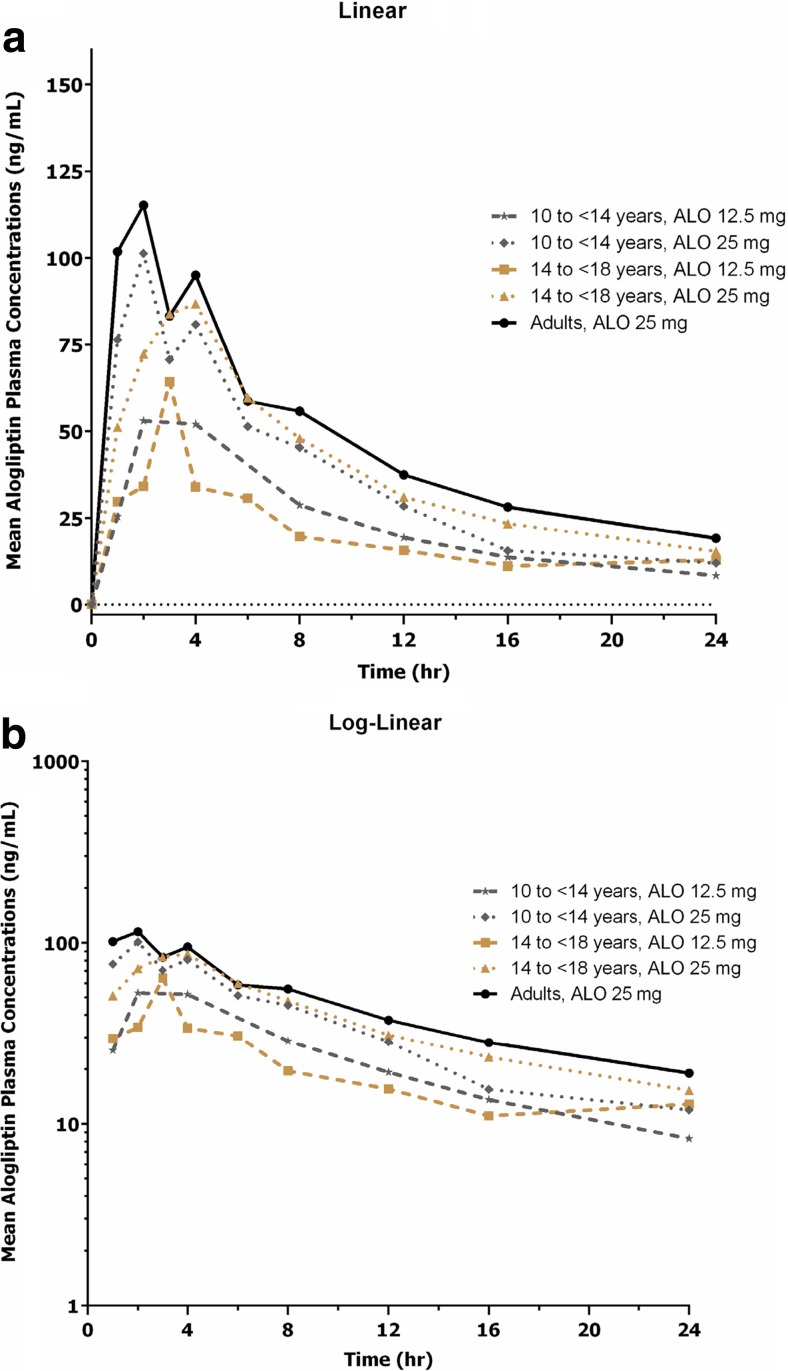
The **a** linear and **b** log-linear plots of mean plasma concentrations of alogliptin vs time after a single dose of alogliptin. *ALO,* alogliptin

Following a single oral administration of alogliptin, the mean alogliptin plasma concentrations reached the maximum at 2 to 4 h and gradually declined thereafter through 24 h across all three treatment groups for both 12.5- and 25-mg doses. The linear and log-linear plots of mean plasma concentrations of alogliptin vs time after a single dose of alogliptin are shown in Fig. 1.

**Fig. 2:**
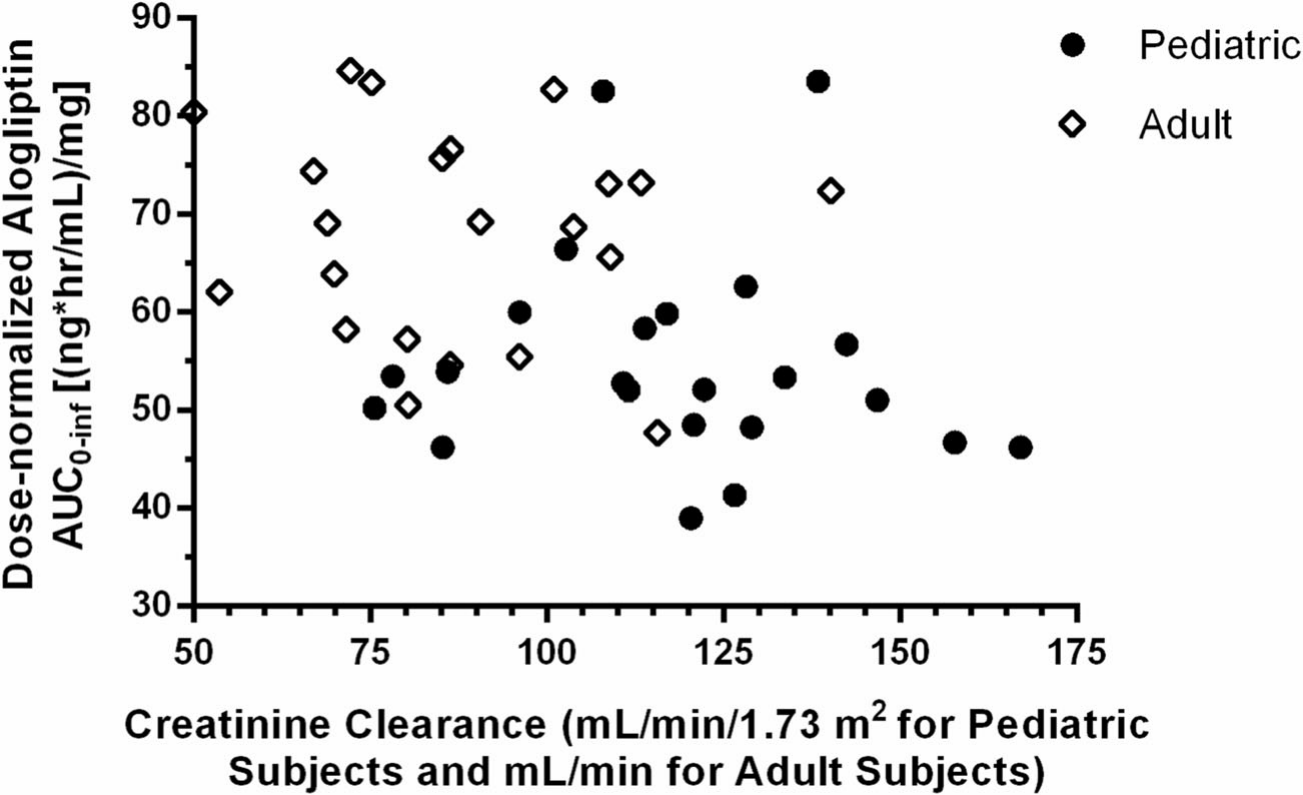
Scatter plots of dose-normalized AUC_0-inf_ values of alogliptin in children, adolescents, and adults with type 2 diabetes mellitus vs creatinine clearance. *AUC*_*0-inf,*_ area under the plasma concentration-time curve from time 0 to infinity

The median T_max_ values of alogliptin after a single oral administration ranged from 2 to 4 h across all three treatment groups. The mean C_max_ and AUC_0-inf_ values were similar between the two pediatric groups (groups 1 and 2) but were 23 to 29% lower in all pediatric subjects (groups 1 and 2) than in adult subjects for the 25-mg dose. In each pediatric group, C_max_ and AUC_0-inf_ values increased in a near-dose-proportional fashion between the 12.5- and 25-mg doses. The mean CL/F values were similar between the two pediatric groups (groups 1 and 2), but were up to 37% higher in all pediatric subjects (groups 1 and 2) compared with adult subjects. The mean T_1/2_ and Vz/F values were generally similar in pediatric and adult subjects. Except for C_max_ and AUC_0-inf_, mean values for other plasma PK parameters appeared to be generally similar across pediatric groups and dose levels. The mean CLr and Fe values of alogliptin were generally similar in pediatric and adult subjects for both dose levels. The intersubject variability for all alogliptin plasma and urine PK parameters was low (9–55% for plasma and 6–33% for urine). The plasma and urine PK parameters following a single oral administration of alogliptin 12.5 mg or 25 mg in children, adolescents, and adults with T2DM are summarized in Table 2. Regarding the impact of renal function on exposures, no correlations were observed between dose-normalized AUC_0-inf_ values of alogliptin and creatinine clearance (CrCl) in pediatric and adult subjects, despite the higher CrCl in pediatric subjects (CrCl 75.5–167 mL/min/1.73 m^2^ in pediatric subjects vs CrCl 53.6–140.2 mL/min in adult subjects). Scatter plots of dose-normalized AUC_0-inf_ values of alogliptin in children, adolescents, and adults with T2DM vs CrCl are shown in Fig. 2.

**Table 2:** The plasma and urine pharmacokinetic parameters following a single oral administration of alogliptin 12.5 or 25 mg in children, adolescents, and adults with type 2 diabetes mellitus

Treatment	Group	Number	Statistic	C_max_ (ng/mL)	T_max_ (hr)^b,c^	AUC_0-inf_ (ng·hr./mL)	CL/F (L/hr)	Vz/F (L)	T_1/2_ (hr)	CLr (L/hr)	Fe (%)
ALO 12.5 mg	10 to <14 years	5^a^	Mean %CV	57.8 55	4.00 2.00, 4.08	789.3 18	16.21 16	387.7 20	16.75 19	11.6 24	60.7 13
	14 to <18 years	7	Mean %CV	44.2 38	3.00 1.00, 23.97	689.0 27	19.18 23	426.0 26	15.38 12	14.4 33	54.5 20
ALO 25 mg	10 to <14 years	4	Mean %CV	101.4 23	2.04 2.00, 2.08	1222.0 10	20.65 12	543.4 22	18.09 10	14.5 19	59.2 12
	14 to <18 years	7	Mean %CV	96.7 29	3.97 1.00, 4.08	1318.0 9	19.11 10	468.7 19	17.15 21	13.4 19	61.0 6
	Adults	22	Mean %CV	136.5 25	2.00 1.00, 4.07	1704.0 16	15.06 17	420.8 25	19.33 17	11.4 19	60.1 14

*ALO,* alogliptin; *AUC*_*0-inf,*_ area under the plasma concentration-time curve from time 0 to infinity; *CL/F,* apparent clearance after oral administration; *CLr,* renal clearance from 0 to 24 h postdose; *C*_*max,*_ maximum observed plasma concentration; *CV,* coefficient of variation; *Fe,* fraction of drug excreted in urine from 0 to 36 h postdose; *T*_*1/2,*_ terminal elimination half-life; *T*_*max,*_ time to reach C_max_; *Vz/F,* apparent volume of distribution

^a^ *N* = 4 for CLr and Fe

^b^Median is presented for T_max_ instead of mean

^c^Minimum, maximum is presented for T_max_ instead of %CV

### PD data

**Fig. 3:**
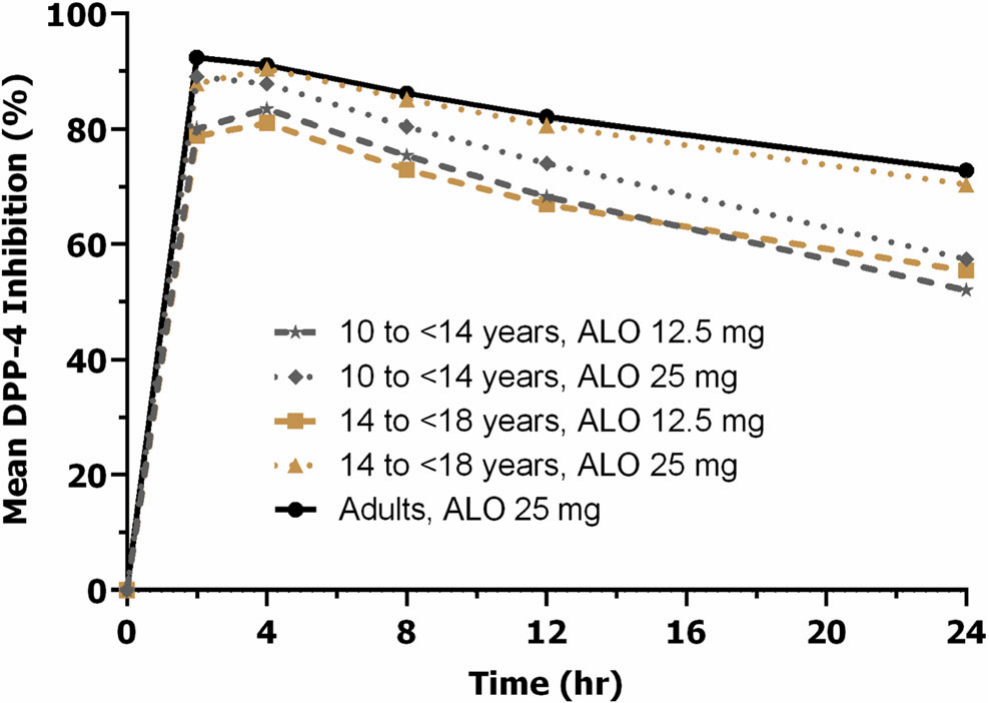
The mean DPP-4 inhibition vs time following a single dose of alogliptin. *ALO,* alogliptin; *DPP-4,* dipeptidyl peptidase-4

Administration of alogliptin resulted in rapid DPP-4 inhibition in both pediatric and adult subjects with T2DM. The DPP-4 inhibition effect of alogliptin peaked at 2 to 4 h, reaching more than 80% inhibition, and gradually declined to at least 50% at 24 h across all three treatment groups for both the 12.5- and 25-mg doses. The mean DPP-4 inhibition vs time following a single dose of alogliptin is shown in Fig. 3.

The median time to E_max_ after a single dose of alogliptin ranged from 2 to 4 h across all three treatment groups. The mean values for E_max_ were similar between the pediatric and adult subjects for the 25-mg dose. Mean values for AUEC_0–24_ and E_24_ were lower in all pediatric subjects than in the adult subjects. Within pediatric groups, the mean values for E_max_ were lower for the 12.5-mg dose (approximately 80%) than for the 25-mg dose (approximately 90%); the mean values for AUEC_0–24_ were approximately 8 and 19% lower for the 12.5-mg dose than for the 25-mg dose in group 1 and group 2, respectively. Within each dose level, DPP-4 inhibition appeared to be generally similar between groups 1 and 2. The intersubject variability for PD parameters of DPP-4 inhibition was very low (≤20%). The PD parameters of DPP-4 inhibition following a single oral administration of alogliptin 12.5 or 25 mg in children, adolescents, and adults with T2DM are summarized in Table 3.

**Table 3:** The pharmacodynamic parameters of DPP-4 inhibition following a single oral administration of alogliptin 12.5 or 25 mg in children, adolescents, and adults with type 2 diabetes mellitus

Treatment	Group	Number	Time to E_max_ (hr)	E_max_ (%)	AUEC_0–24_ (%·hr)	E_24_ (%)
			Median (Min, Max)	Mean (%CV)	Mean (%CV)	Mean (%CV)
ALO 12.5 mg	10 to <14 years	5	4.05 (2.00, 4.08)	83.7 (5)	1570 (7)	52.0 (20)
	14 to <18 years	7	4.00 (2.00, 4.03)	81.6 (7)	1558 (12)	55.4 (16)
ALO 25 mg	10 to <14 years	4	2.08 (2.00, 4.00)	89.3 (3)	1699 (4)	57.4 (9)
	14 to <18 years	7	4.00 (3.97, 4.12)	90.4 (2)	1854 (3)	70.4 (8)
	Adults	22^a^	2.00 (2.00, 4.07)	92.7 (2)	1890 (4)	72.8 (7)

*ALO,* alogliptin; *AUEC*_*0–24*_, area under the plasma effect-time curve from time 0 to 24 h postdose; *CV,* coefficient of variation; *DPP-4*, dipeptidyl peptidase-4; *E*_*24*_, observed effect at 24 h postdose; *E*_*max*_, maximum observed effect

^a^ *N* = 21 for E_24_

### PK/PD modeling and simulations

The PK/PD model consists of a two-compartment model with first-order absorption and elimination and body weight effects on clearance and volume parameters, plus a direct E_max_ model linking plasma alogliptin concentrations to DPP-4 inhibition. This model adequately described the PK/PD data in both pediatric and adult subjects with T2DM, as demonstrated by the goodness-of-fit test and visual predictive check (Supplemental Figs. 1 and 2). The model parameters were estimated with good precision (Supplemental Table 1). Although separate values for absorption rate constant, oral clearance, and EC_50_ were reported for pediatric and adult subjects, the differences were not considered clinically relevant. Except for body weight, no additional covariates were retained in the model.

**Fig. 4:**
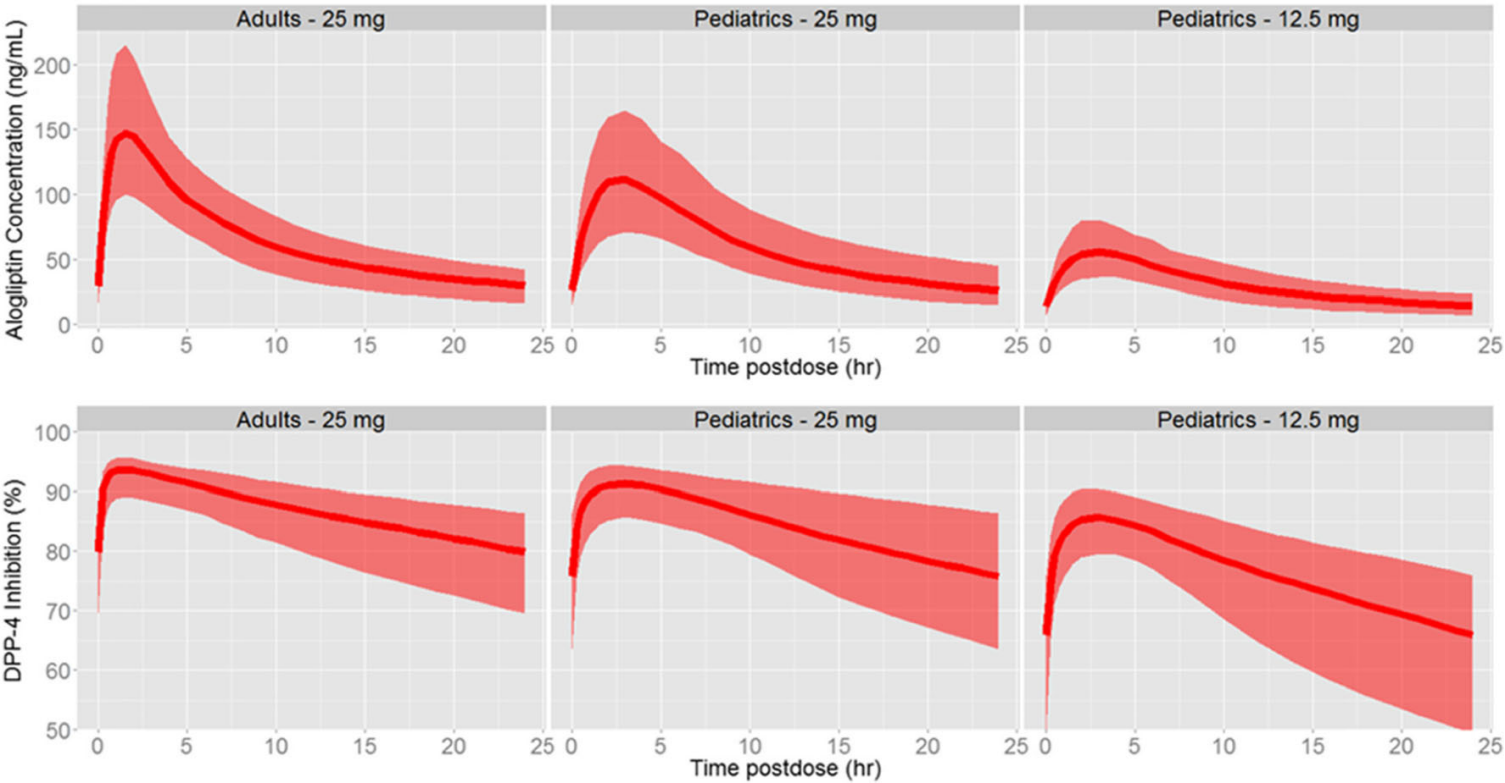
The simulated alogliptin plasma concentrations and DPP-4 inhibition vs time after a single dose of alogliptin. *DPP-4*, dipeptidyl peptidase-4. The *solid line* is the median; the *shaded region* is the 90% prediction interval

Following repeated doses of alogliptin, the simulated C_max_ and AUC_0-tau_ values of alogliptin 25 mg were higher than those of alogliptin 12.5 mg in adult and pediatric subjects. The simulated adult exposure was consistent with that from a phase 1 study in adult subjects with T2DM receiving 25 mg alogliptin [10]. The simulated C_max_ and AUC_0-tau_ values were generally lower in pediatric subjects than in adult subjects. Despite these modest differences in exposure to alogliptin, the simulated E_max_ values were similar between pediatric and adult subjects, which is consistent with the study results and previously reported PD activity in adult T2DM subjects [10]. Within pediatric subjects, simulated DPP-4 inhibition levels at steady state through 24 h postdose were 65.2 and 75.1% following alogliptin 12.5 and 25 mg doses, respectively. The simulated alogliptin plasma concentrations and DPP-4 inhibition vs time after repeated doses of alogliptin are shown in Fig. 4. The simulated plasma PK and PD parameters following repeated oral administrations of alogliptin are summarized in Table 4.

**Table 4:** The simulated plasma pharmacokinetic and pharmacodynamic parameters following repeated oral administrations of alogliptin

Group	Dose (mg)	C_max_ (ng/mL)	AUC_0-tau_ (ng·hr/mL)	E_24_ (%)
Pediatric	12.5	55.6	729.5	66.0
	25	112.2	1400.2	75.8
Adult^a^	12.5	73.4	777.2	70.5
	25	147.7	1575.1	79.9

*AUC*_*0-tau*_ area under the plasma concentration-time curve from time 0 to the end of the dosing period; *C*_*max*_, maximum observed plasma concentration; *E*_*24*_, observed effect at 24 h postdose

^a^In the phase 1 study in adults with T2DM, the C_max_ was 152.8 ng/mL, AUC_0-tau_ was 1473.7 ng·h/mL, and E_24_ was 81.8%

### Safety

During the entire study period, 20 subjects experienced 36 treatment-emergent AEs (TEAEs; Table 5). Most of the TEAEs were mild. One moderate (joint swelling) AE was experienced by one subject, and one severe (headache) AE was experienced by one subject in group 3 receiving alogliptin 25 mg; both were considered by the investigators to be unrelated to the study drug. The most frequently reported TEAEs (occurred in ≥2 subjects in any dose level in any group) were headache, nausea, fatigue, and abdominal pain. No AEs led to study drug discontinuation and no deaths occurred. An overview of TEAEs is provided in Table 5.

**Table 5:** Overview of TEAEs

	Group 1 10 to <14 years				Group 2 14 to <18 years				Group 3 Adults	
	Alogliptin 12.5 mg *N* = 5		Alogliptin 25 mg *N* = 4		Alogliptin 12.5 mg *N* = 8		Alogliptin 25 mg *N* = 7		Alogliptin 25 mg *N* = 22	
	Events, *N*	Subjects, *N* (%)	Events, *N*	Subjects, *N* (%)	Events, *N*	Subjects, *N* (%)	Events, *N*	Subjects, *N* (%)	Events, *N*	Subjects, *N* (%)
TEAEs^a,b^	3	3 (60.0)	1	1 (25.0)	9	5 (62.5)	3	2 (28.6)	20	9 (40.9)
Related^b^	2	2 (40.0)	0	0	2	2 (25.0)	0	0	8	5 (22.7)
Not related	1	1 (20.0)	1	1 (25.0)	7	3 (37.5)	3	2 (28.6)	12	4 (18.2)
Mild^c^	3	3 (60.0)	1	1 (25.0)	9	5 (62.5)	3	2 (28.6)	18	7 (31.8)
Moderate^c^	0	0	0	0	0	0	0	0	1	1 (4.5)
Severe^c^	0	0	0	0	0	0	0	0	1	1 (4.5)

*TEAE*, treatment emergent adverse event

^a^Includes TEAEs considered by the investigator to be possibly, probably, or definitely related to the study drug

^b^If a subject had related and not-related TEAEs, the subject was counted only as having related TEAEs

^c^If a subject had TEAEs of different intensities, the subject was counted only for the most extreme TEAE

One postdose abnormal serum chemistry test result (increased blood creatine kinase) in one subject (group 2 receiving alogliptin 25 mg), two postdose abnormal hematology test results in two subjects (decreased neutrophil count in group 1 receiving alogliptin 12.5 mg and decreased hematocrit and hemoglobin in group 2 receiving alogliptin 25 mg), and one postdose abnormal vital sign result (increased body temperature) in one subject (group 1 receiving alogliptin 12.5 mg) were reported as TEAEs. All these TEAEs were mild in intensity. No abnormal ECG result was reported as a TEAE.

## Discussion

Following a single oral administration of alogliptin 12.5 or 25 mg tablets to children, adolescents, and adults with T2DM, the plasma and urine PK of alogliptin appeared to be generally similar between the two age groups of pediatric subjects. Within pediatric subjects, dose-proportional increases in mean C_max_ and AUC_0-inf_ values of alogliptin were observed between the 12.5- and 25-mg doses. The median T_max_ values were similar across the pediatric and adult groups, indicating similar rates of appearance of alogliptin in plasma. The mean C_max_ and AUC_0-inf_ values of alogliptin were 23 to 29% lower in all pediatric subjects than in adult subjects for the alogliptin 25 mg dose, suggesting the extent of exposure was lower in pediatric subjects than in adult subjects. Because CrCl, a measure of renal function, was higher in pediatric subjects (CrCl 75.5–167 mL/min/1.73 m^2^) than in adult subjects (CrCl 53.6–140.2 mL/min), the slight decreases in C_max_ and AUC_0-inf_ in pediatric subjects may be due to the slightly higher renal function in these subjects. The mean CL/F values of alogliptin were up to 37% higher in pediatric subjects than in adult subjects. The other plasma and urine PK parameters of alogliptin (Vz/F, T_1/2_, Clr, Fe) were generally similar across the pediatric and adult subject groups.

Administration of alogliptin resulted in rapid DPP-4 inhibition in both pediatric and adult subjects with T2DM. Although the extent of exposure was lower in pediatric subjects than in adult subjects as demonstrated by the mean C_max_ and AUC_0-inf_ values, the levels of DPP-4 inhibition—indicated by the mean E_max_ and AUEC_0–24_ values—were similar between the two populations. This is particularly true for the adolescent group (age 14 to <18 years), in which the E_max_ and AUEC_0–24_ values were almost identical to those in the adult group. Within pediatric subjects, the mean E_max_ values were approximately 80 and 90% for alogliptin 12.5 and 25 mg, respectively; the mean AUEC_0–24_ values were approximately 8 and 19% lower for the 12.5-mg dose than for the 25-mg dose in group 1 and group 2, respectively. Within each dose level, DPP-4 inhibition appeared to be generally similar in groups 1 and 2. Model-simulated alogliptin PK and PD parameters were similar to those observed in this study. Single doses of alogliptin 12.5 and 25 mg were well tolerated in pediatric and adult subjects. No dose relationship was observed in the percentages of pediatric subjects who had TEAEs, and the incidence of TEAEs was lower in pediatric subjects who received alogliptin 25 mg than in adult subjects.

The majority of the study subjects were African American; pediatric T2DM is particularly prevalent among African Americans [2], which limited the availability of subjects of other races. Since race has no clinically meaningful effect on alogliptin PK in adults [9], our study results are likely to be applicable to pediatric patients of other races. However, this speculation needs to be confirmed in future larger pediatric trials including more non-African American subjects.

This study excluded subjects with renal impairment or hypertension, two common comorbidities of T2DM. Because alogliptin primarily relies on renal excretion [20], renal function is a major determinant of drug exposure. In a PK study in adults, exposure to alogliptin increased approximately 1.7-fold in patients with mild renal impairment, 2.1-fold in patients with moderate renal impairment, and 3.8-fold in patients with end stage renal disease (ESRD), relative to healthy subjects [20]. Therefore, alogliptin prescribing information recommends a 50% dose reduction for adult patients with moderate renal impairment (CrCl ≥30 to <60 mL/min) and a further 50% dose reduction for patients with severe renal impairment (CrCl ≥15 to <30 mL/min) or ESRD (CrCl <15 mL/min or requiring hemodialysis) [9]. However, in clinical studies, alogliptin displayed similar efficacy and safety profiles between patients with renal impairment and normal renal function [9]. Additionally, this study did not evaluate the effect of hypertension on the PD of alogliptin. In contrast to renal function, an experimentally proven determinant of drug exposure, hypertension is not expected to have a significant influence on dose setting and, therefore, has not been extensively studied. There is very little information regarding the effect of hypertension on PD of alogliptin. However, alogliptin was shown to lower blood pressure in patients with T2DM [21].

In addition, in an alogliptin cardiovascular safety trial in which 83% of patients had hypertension, alogliptin demonstrated a similar rate of major adverse cardiovascular events to that of placebo in these patients [9]. Given the minor impact of hypertension on alogliptin exposure and safety and the low incidence of this condition among children, not assessing the effect of hypertension on DPP-4 inhibition is unlikely to affect the purpose of this study. As a phase 1 study and the first study of alogliptin in pediatric patients, this study had a small sample size. The purpose of the study was to establish a basic dose for future phase 3 pediatric studies, and the inclusion criteria only allowed pediatric subjects without clinically significant disorders other than T2DM and adult subjects without clinically significant disorders other than T2DM and controlled hypertension. As such, eliminating the interferences of comorbidities on alogliptin PK profile maintains analysis sensitivity for this small study. Based on adult PK data, we speculate that renal function may have a similar impact on alogliptin PK in children. This needs to be tested in future larger pediatric studies including subjects with renal impairment.

Based on the PK and PD data and model simulations, pediatric subjects with T2DM require the 25-mg dose of alogliptin to achieve alogliptin exposures and DPP-4 inhibition similar to those in adults with T2DM. The 25-mg dose in adolescents best approximates the DPP-4 inhibition in adults following 25-mg dose administration, despite some modest differences in alogliptin PK (simulated median steady state C_max_ and AUC_0-tau_ values in adolescents was 24 and 11% lower, respectively, relative to adults) between the two populations. The DPP-4 inhibition in adolescents receiving a 12.5-mg dose was considered suboptimal, since efficacy requires steady state trough DPP-4 inhibition of approximately 80% [22]. Therefore, the 25-mg dose that has shown to be safe and efficacious in adults should be a suitable dose for evaluation in the pediatric phase 3 program. It should be noted, however, that alogliptin is not approved for use in pediatric patients with T2DM.

## Electronic supplementary material

ESM 1 (DOCX 16 kb)

Supplemental Fig. 1 (DOCX 274 kb)

Supplemental Fig. 2 (DOCX 404 kb)

Supplemental Table 1 (DOCX 14 kb)
