# Supplementary material for: The pharmacokinetics and pharmacodynamics of alogliptin in children, adolescents, and adults with type 2 diabetes mellitus
Source: Eur J Clin Pharmacol. 2016 Dec 20;73(3):279–88. doi: 10.1007/s00228-016-2175-1 (PMC5306220; doi:10.1007/s00228-016-2175-1)
Supplement: Supplementary file 1 — (DOCX 16 kb) [file 228_2016_2175_MOESM1_ESM.docx]

**European Journal of Clinical Pharmacology**

**The Pharmacokinetics and Pharmacodynamics of Alogliptin in Children, Adolescents, and Adults with Type 2 Diabetes Mellitus**

Caroline Dudkowski, Max Tsai, Jie Liu, Zhen Zhao, Eric Schmidt, Jeannie Xie

Takeda Development Center Americas, Inc.

One Takeda Parkway

Deerfield, IL 60015

Corresponding author:

Caroline Dudkowski

Takeda Pharmaceuticals U.S.A., Inc.

One Takeda Parkway

Deerfield, IL 60015

Email: caroline.dudkowski@takeda.com

Telephone: 224-554-2005

Fax: 224-554-7933

**Subject enrollment criteria**

All subjects were required to have a diagnosis of non–insulin-dependent type 2 diabetes mellitus (T2DM) based on the diagnostic criteria of the American Diabetes Association (further confirmed by C-peptide measurement) and were allowed to take concomitant metformin if the dose was stable for ≥30 days prior to day 1 [1]. In addition, included children and adolescent subjects weighed ≥36 kg (79 pounds), had a screening body mass index (BMI) of ≥18 kg/m2, and had a fasting serum C-peptide concentration ≥0.8 ng/mL (≥0.26 nmol/L) at screening visit. Included adult subjects weighed ≥50 kg (110 pounds), had a screening BMI between 23 and 45 kg/m2 (20–35 kg/m2 for Asian subjects), and could be taking statin or antihypertensive drugs if the dose was stable for ≥30 days prior to study day 1.

Individuals were not permitted to enroll if they had a hypersensitivity to alogliptin or related compounds; a history of type 1 diabetes or secondary forms of diabetes, including maturity onset diabetes of the young; hemoglobin value <12 g/dL; systolic blood pressure >140 mm Hg or diastolic blood pressure >90 mm Hg; alanine aminotransferase (ALT) or aspartate aminotransferase (AST) levels greater than 2 times (1.5 times for pediatric subjects) the upper limit of normal (ULN), active liver disease, or jaundice; serum creatinine level >1.5 mg/dL; creatinine clearance <50 mL/min; or treatment with ketoconazole, fluconazole, gemfibrozil, rifampin, or carbamazepine before or during the study.

**PK/PD analysis precision and accuracy**

For alogliptin plasma concentration measurements, the quantification ranges for plasma and urine samples were 1.00 to 250 ng/mL and 5.00 to 5000 ng/mL, respectively. Precision and accuracy were evaluated by replicate analyses of plasma or urine quality control (QC) pools prepared at 5 concentrations spanning the calibration range. Precision was measured as the percent coefficient of variation (%CV) of the set of values for each pool. Accuracy was expressed as the percent difference of the mean value for each pool from the theoretical concentration. For the plasma QC concentrations of 2.50 ng/mL, 5.00 ng/mL, 15.0 ng/mL, 45.0 ng/mL, and 200 ng/mL, the correlating %CV were 7.53, 4.78, 4.13, 3.86, and 1.85, respectively; and the correlating percentages of difference from the theoretical concentrations were 4.34, 1.71, 1.10, 0.146, and −0.235, respectively. For the urine QC concentrations of 12.5 ng/mL, 50 ng/mL, 250 ng/mL, 800 ng/mL, and 4000 ng/mL, the correlating %CV were 4.03, 4.53, 3.67, 3.85, and 4.40, respectively; and the correlating percentages of difference from the theoretical concentrations were 1.84, −1.16, 0.689, −0.278, and −0.812, respectively.

For DPP-4 inhibition measurements, the QC samples were prepared as low-, medium-, and high-DPP-4 activity levels by fortifying blank matrix with alogliptin at concentrations of 500 nM, 50.0 nM, and 5.00 nM, respectively. Results for study samples are expressed as percent inhibition, while results for QCs are expressed as percent activity. Precision was evaluated by analyzing for percent activity in a positive control (blank matrix), a negative control (blank buffer), and low-, medium-, and high-activity QCs. Precision was expressed as overall %CV for the replicate percent activity values at each control level. For acceptable sets of DPP-4 inhibition measurements, the %CV correlating the positive control, the negative control, QC 5.00 nM, QC 50.0 nM, and QC 500 nM were 3.64, −1757, 8.33, 11.8, and 11.8, respectively.

**References**

1. American Diabetes Association. Standards of Medical Care in Diabetes—2009. Diabetes Care 32 (Supplement 1)
