# Supplementary material for: The pharmacokinetics and pharmacodynamics of alogliptin in children, adolescents, and adults with type 2 diabetes mellitus
Source: Eur J Clin Pharmacol. 2016 Dec 20;73(3):279–88. doi: 10.1007/s00228-016-2175-1 (PMC5306220; doi:10.1007/s00228-016-2175-1)

**European Journal of Clinical Pharmacology**

**The Pharmacokinetics and Pharmacodynamics of Alogliptin in Children, Adolescents, and Adults with Type 2 Diabetes Mellitus**

Caroline Dudkowski, Max Tsai, Jie Liu, Zhen Zhao, Eric Schmidt, Jeannie Xie

Takeda Development Center Americas, Inc.

One Takeda Parkway

Deerfield, IL 60015

Corresponding author:

Caroline Dudkowski

Takeda Pharmaceuticals U.S.A., Inc.

One Takeda Parkway

Deerfield, IL 60015

Email: caroline.dudkowski@takeda.com

Telephone: 224-554-2005

Fax: 224-554-7933

**Supplemental Fig. 1** Goodness-of-Fit Plots for Final Pharmacokinetic (upper panel) and Pharmacodynamic (lower panel) Model
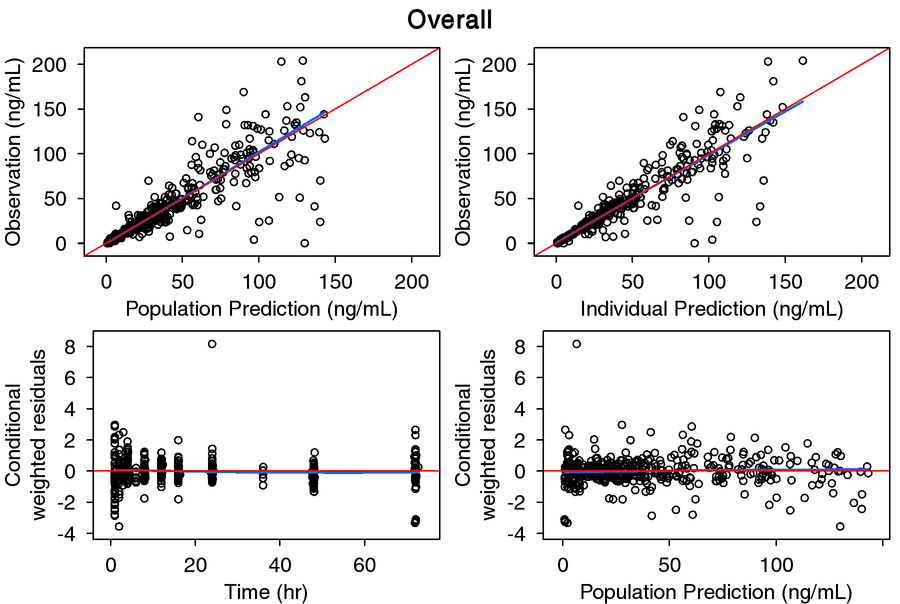


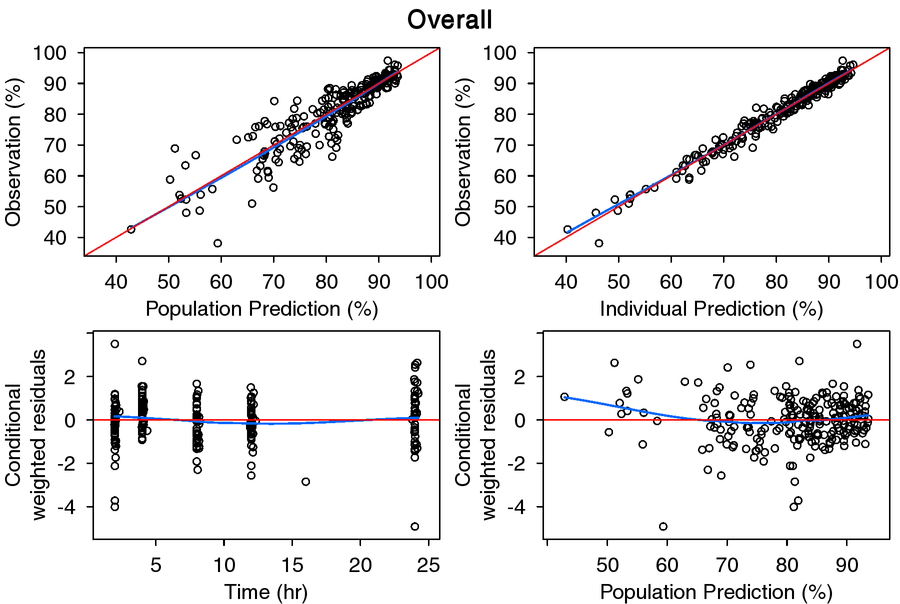

Supplement: Supplementary file 2 — (DOCX 274 kb) [file 228_2016_2175_MOESM2_ESM.docx]
