# Supplementary material for: The pharmacokinetics and pharmacodynamics of alogliptin in children, adolescents, and adults with type 2 diabetes mellitus
Source: Eur J Clin Pharmacol. 2016 Dec 20;73(3):279–88. doi: 10.1007/s00228-016-2175-1 (PMC5306220; doi:10.1007/s00228-016-2175-1)
Supplement: Supplementary file 4 — (DOCX 14 kb) [file 228_2016_2175_MOESM4_ESM.docx]

**European Journal of Clinical Pharmacology**

**The Pharmacokinetics and Pharmacodynamics of Alogliptin in Children, Adolescents, and Adults with Type 2 Diabetes Mellitus**

Caroline Dudkowski, Max Tsai, Jie Liu, Zhen Zhao, Eric Schmidt, Jeannie Xie

Takeda Development Center Americas, Inc.

One Takeda Parkway

Deerfield, IL 60015

Corresponding author:

Caroline Dudkowski

Takeda Pharmaceuticals U.S.A., Inc.

One Takeda Parkway

Deerfield, IL 60015

Email: caroline.dudkowski@takeda.com

Telephone: 224-554-2005

Fax: 224-554-7933

**Supplemental Table 1.** Model Parameter Estimates.

| Parameter | Final Parameter Estimate | | Interindividual Variability/Residual Variability | |
| --- | --- | --- | --- | --- |
|  | Typical Value | % SEM | Magnitude | % SEM |
| Absorption rate constant [KA] in pediatric subjects (1/hr) | 0.590 | 19.0 | NE | NE |
| Oral clearance [CL/F] in pediatric subjects (L/hr) | 14.4 | 4.86 | 18.5 %CV | 53.7 |
| Central volume of distribution [V2/F] (L) | 125 | 8.83 | 30.1 %CV | 27.6 |
| Peripheral volume of distribution [V3/F] (L) | 117 | 7.16 | 0 %CV | FIXED |
| Intercompartmental clearance [Q/F] (L/hr) | 11.7 | 21.3 | 0 %CV | FIXED |
| Baseline response [E0] (%) | 0 | FIXED | 0 %CV | FIXED |
| Maximal response [E_max_] (%) | 99.3 | 1.78 | 0 %CV | FIXED |
| Concentration at half-maximal response [EC50] in pediatric subjects (ng/mL) | 6.38 | 8.64 | 24.6 %CV | 26.3 |
| Shape factor [γ] | 0.857 | 10.1 | 0 %CV | FIXED |
| Concentration at half-maximal response [EC50] in adult subjects (ng/mL) | 5.45 | 8.13 | 24.6 %CV | 26.3 |
| Oral clearance [CL/F] in adult subjects (L/hr) | 13.0 | 3.53 | 18.5 %CV | 53.7 |
| Absorption rate constant [KA] in adult subjects (1/hr) | 1.66 | 41.2 | NE | NE |
| PK Proportional Residual Variability | 0.0820 | 25.2 | 28.6 %CV | NA |
| PK Additive Residual Variability | 0.0100 | FIXED | 0.100 SD | NA |
| PD Additive Residual Variability | 3.67 | 18.0 | 1.92 SD | NA |
| Minimum value of the objective function = 2725.152 | | | | |

CV, coefficient of variation; NA, not available; NE, not estimated; PD, pharmacodynamic; PK, pharmacokinetic; SD, standard deviation; SEM, standard error of the mean.
